# A Novel Ambisense Densovirus, *Acheta domesticus* Mini Ambidensovirus, from Crickets

**DOI:** 10.1128/genomeA.00914-13

**Published:** 2013-11-07

**Authors:** Hanh T. Pham, Qian Yu, Max Bergoin, Peter Tijssen

**Affiliations:** INRS-Institut Armand-Frappier, Laval, QC, Canada

## Abstract

The genome structure of *Acheta domesticus* mini ambidensovirus, isolated from crickets, resembled that of ambisense densoviruses from *Lepidoptera* but was 20% smaller. It had the highest (<25%) protein sequence identity with the nonstructural protein 1 (NS1) of *Iteravirus* and VP of *Densovirus* members (both with 25% coverage) and smaller (0.2- versus 0.55-kb) Y-shaped inverted terminal repeats.

## GENOME ANNOUNCEMENT

The cricket industry has been devastated worldwide recently by the *Acheta domesticus* densovirus (AdDNV) (1–4). We also observed several, thus far unknown, viruses such as volvoviruses, which have circular, single-stranded DNA (ssDNA) genomes (5), and a new densovirus (parvovirus).

Two genera of insect parvoviruses, named densoviruses (6), are particularly relevant for this new densovirus. The *Densovirus* genus contains ambisense densoviruses from *Lepidoptera*, with genomes of 6 kb, Y-shaped inverted terminal repeats (ITRs) of about 0.55 kb, and sequence identities of about 85% (7–11). The *Iteravirus* genus contains monosense densoviruses, also from *Lepidoptera*, with 5-kb genomes, J-shaped 0.25-kb inverted terminal repeats (ITRs), and about 75% sequence identities (12–15).

A new virus with morphology and size similar to densoviruses was detected in some cricket samples from the United States. Virus was purified and DNA extracted as described previously (5). Digestion of viral DNA with EcoRI yielded 2 bands of about 700 bp and 4,200 bp on agarose gels. DNA was blunt ended with T4 DNA polymerase and a large Klenow fragment in the presence of dNTPs at room temperature (RT), ligated into the EcoRV site of pBlueskript KS(+), and transformed into SURE cells. DNA of clones with expected sizes were subcloned. Digestion with EcoNI within the terminal hairpins yielded clear reads of ITR sequences. Several complete clones were sequenced in both directions by use of Sanger’s primer-walking method as described previously (11). Contigs were assembled by use of the CAP3 program (http://pbil.univ-lyon1.fr/cap3.php/) (16).

Surprisingly, the genome structure and gene organization of this virus strongly resembled those of ambisense densoviruses from the *Densovirus* genus (7–11), but the genome sequence was only 4,945 nucleotides (nt) long, instead of about 6,035 nt, and lacked nucleotide sequence identity (best E value of 0.017, with a query coverage of 1%). Protein sequence identities were for the major nonstructural protein 1 (NS1) closest to *Iteravirus* members and, oddly, for the structural proteins (VP) closest to *Densovirus* members (both at best 25% identity for 25% coverage [or higher for shorter coverage]).

ITRs of AdMADV were smaller than those of densovirus members (199 versus about 545 nt) and Y-shaped, with a 113-nt hairpin. The 45-nt-long stem contained two side arms in the middle, nt 46 to 68, that occurred in two sequence orientations (flip/flop). It had a high GC content (63%) and contained inboard TATA boxes, at 193 to 199 for the NS cassette and at 4747 to 4753 for the VP cassette. This structure is identical to that of *Densovirus* ITRs.

The NS cassette consisted, as for *Densovirus* members, of NS3, followed by NS1 and an overlapping NS2. Splicing, as for *Densovirus*, would remove the NS3 open reading frame (ORF) and allow expression of NS1 and NS2 by leaky scanning. As for *Densovirus*, the putative splice acceptor site was located just upstream of the initiation codon of NS1 (1172-CAG/aATG_NS1_..N_19_..ATG_NS2_) (in GmDNV, 1395-CAG/ATG_NS1_..N_4_..ATG_NS2_). As for members of the *Densovirus* genus, the VP on the complementary strand also contained the phospholipase A2 motif (4,590 to 4,680 nt) (17) and the stop codons of NS1 and VP were neighbors (2661-TAG/AAT-2666), suggesting a small overlap of their transcripts, as for GmDNV.

### Nucleotide sequence accession number.

The GenBank accession number of AdMADV is KF275669.
